# Supplementary material for: Assessment of the Antioxidant and Antimicrobial Potential of Ptychotis verticillata Duby Essential Oil from Eastern Morocco: An In Vitro and In Silico Analysis
Source: Antibiotics (Basel). 2023 Mar 27;12(4):655. doi: 10.3390/antibiotics12040655 (PMC10135233; doi:10.3390/antibiotics12040655)
Supplement: Supplementary file 1 [file antibiotics-12-00655-s001.zip › antibiotics-2259503-supplementary.pdf]

Supplementary file

Assessment of the Antioxidant and Antimicrobial Potential of *Ptychotis verticillata* Duby Essential Oil from Eastern Morocco: An In Vitro and In Silico Analysis

Mohamed Taibi [1,2], Amine Elbouzidi [1], Douaae Ou-Yahia [2], Mohammed Dalli [3,4], Reda Bellaouchi [3], Aziz Tikent [1], Mohammed Roubi [3], Nadia Gseyra [3], Abdeslam Asehraou [3], Christophe Hano [5,*], Mohamed Addi [1,*], Bouchra El Guerrouj [1,2] and Khalid Chaabane [1]

1 Laboratoire d'Amélioration des Productions Agricoles, Biotechnologie et Environnement (LAPABE), Faculté des Sciences, Université Mohammed Premier, Oujda 60000, Morocco; mohamedtaibi9@hotmail.fr (M.T.); amine.elbouzidi@ump.ac.ma (A.E.); tikent.aziz@ump.ac.ma (A.T.); b.elguerrouj@ump.ac.ma (B.E.G.); k.chaabane@ump.ac.ma (K.C.)

2 Centre de l'Oriental des Sciences et Technologies de l'Eau et de l'Environnement (COSTEE), Université Mohammed Premier, Oujda 60000, Morocco; douaae.ouyahia@usmba.ac.ma

3 Laboratory of Bioresources, Biotechnology, Ethnopharmacology and Health, Faculty of Sciences, Mohammed First University, Boulevard Mohamed VI, B.P. 717, Oujda 60000, Morocco; m.dalli@ump.ac.ma (M.D.); r.bellaouchi@ump.ac.ma (R.B.); mohammed.roubi1@ump.ac.ma (M.R.); ngseyra@hotmail.com (N.G.); asehraou@yahoo.fr (A.A.)

4 Laboratory of Microbiology, Faculty of Medicine and Pharmacy, University Mohammed The First, Oujda 60000, Morocco

5 Laboratoire de Biologie des Ligneux et des Grandes Cultures, INRAE USC1328, University of Orleans, CEDEX 2, 45067 Orléans, France

* Correspondence: hano@univ-orleans.fr (C.H.); m.addi@ump.ac.ma (M.A.)

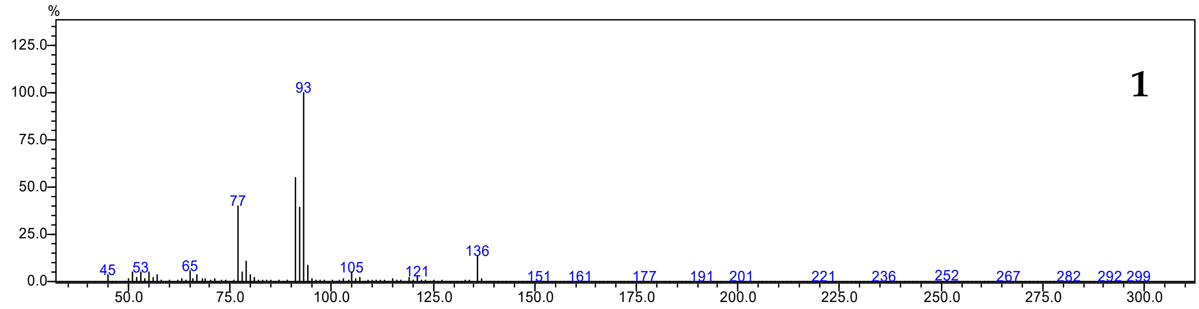
1
%
125.0
100.0
75.0
50.0
25.0
0.0
93
77
136
45 53 65 105 121 151 161 177 191 201 221 236 252 267 282 292 299
50.0 75.0 100.0 125.0 150.0 175.0 200.0 225.0 250.0 275.0 300.0

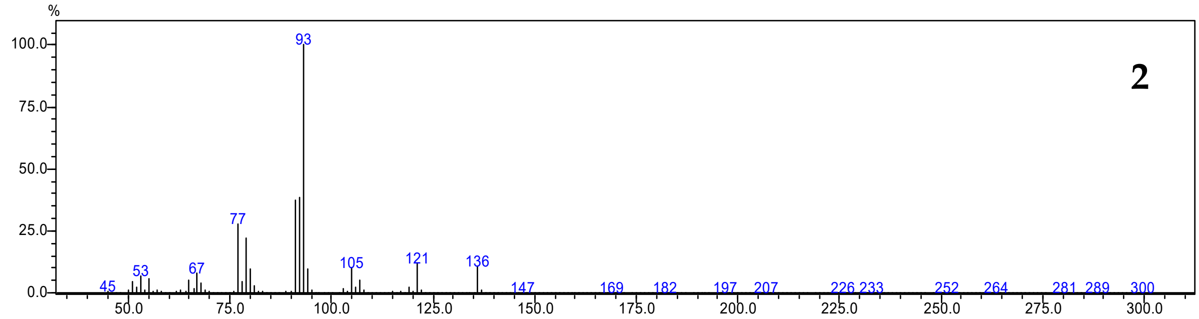
2
%
100.0
75.0
50.0
25.0
0.0
93
77
53 67 105 121 136
45 147 169 182 197 207 226 233 252 264 281 289 300
50.0 75.0 100.0 125.0 150.0 175.0 200.0 225.0 250.0 275.0 300.0

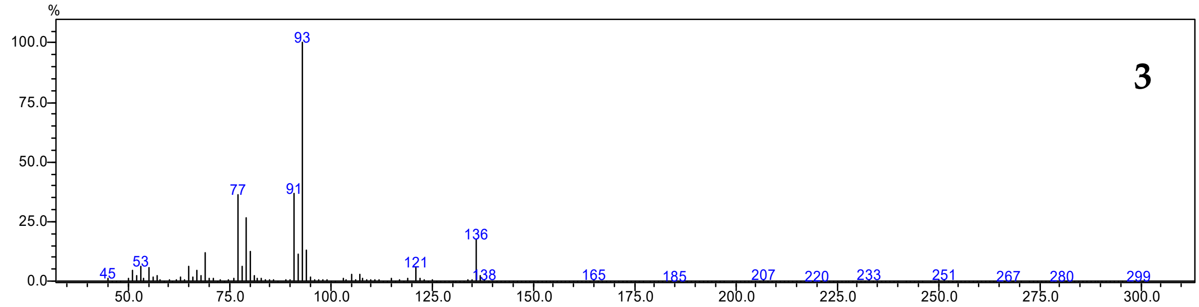
3
%
100.0
75.0
50.0
25.0
0.0
93
77 91
136
53 121
45 138 165 185 207 220 233 251 267 280 299
50.0 75.0 100.0 125.0 150.0 175.0 200.0 225.0 250.0 275.0 300.0

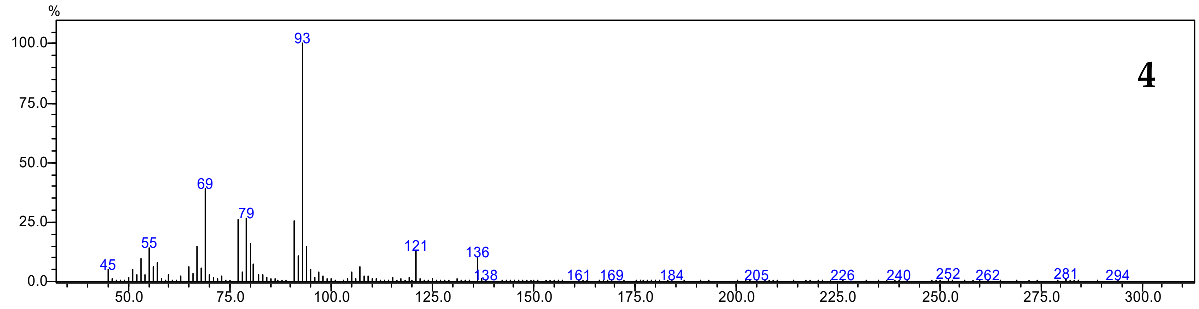
4
%
100.0
75.0
50.0
25.0
0.0
93
69
79
55 121 136
45 138 161 169 184 205 226 240 252 262 281 294
50.0 75.0 100.0 125.0 150.0 175.0 200.0 225.0 250.0 275.0 300.0

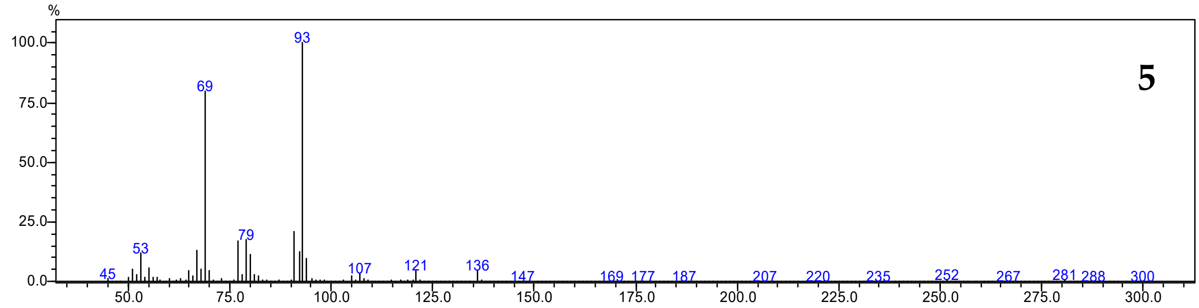
5
%
100.0
75.0
50.0
25.0
0.0
93
69
53 79
45 107 121 136 147 169 177 187 207 220 235 252 267 281 288 300
50.0 75.0 100.0 125.0 150.0 175.0 200.0 225.0 250.0 275.0 300.0

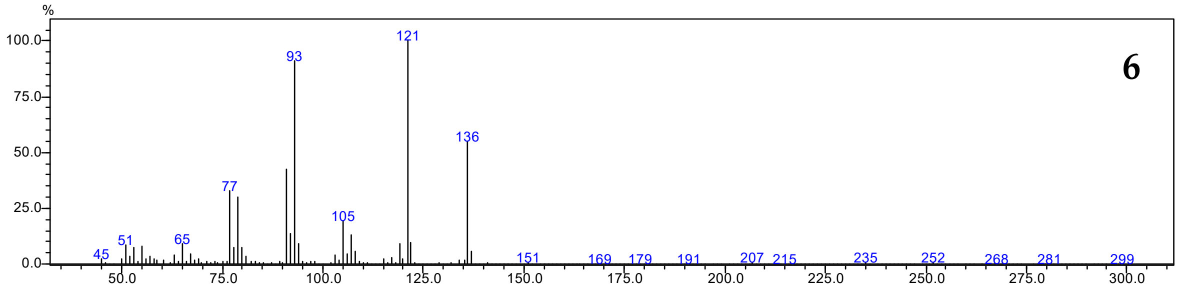
6
%
100.0
75.0
50.0
25.0
0.0
45 51 65 77 93 105 121 136 151 169 179 191 207 215 235 252 268 281 299
50.0 75.0 100.0 125.0 150.0 175.0 200.0 225.0 250.0 275.0 300.0

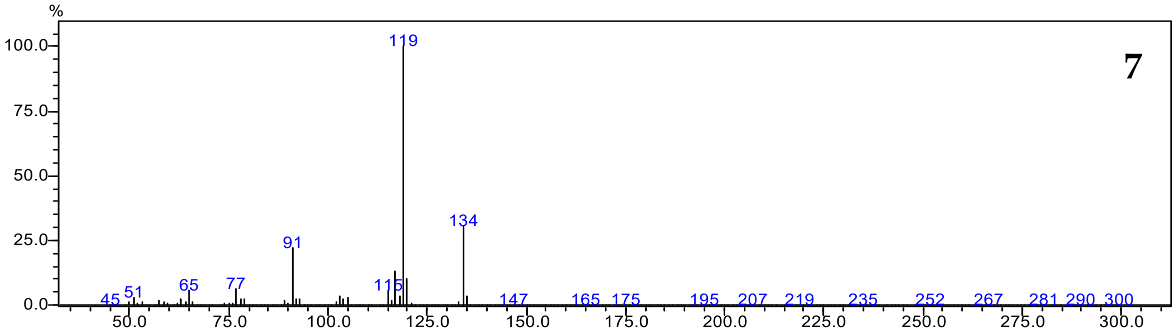
7
%
100.0
75.0
50.0
25.0
0.0
45 51 65 77 91 115 119 134 147 165 175 195 207 219 235 252 267 281 290 300
50.0 75.0 100.0 125.0 150.0 175.0 200.0 225.0 250.0 275.0 300.0

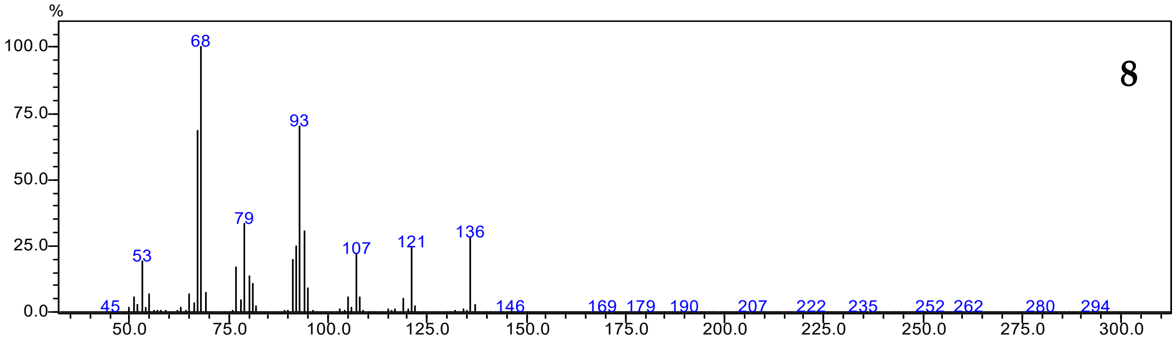
8
%
100.0
75.0
50.0
25.0
0.0
45 53 68 79 93 107 121 136 146 169 179 190 207 222 235 252 262 280 294
50.0 75.0 100.0 125.0 150.0 175.0 200.0 225.0 250.0 275.0 300.0

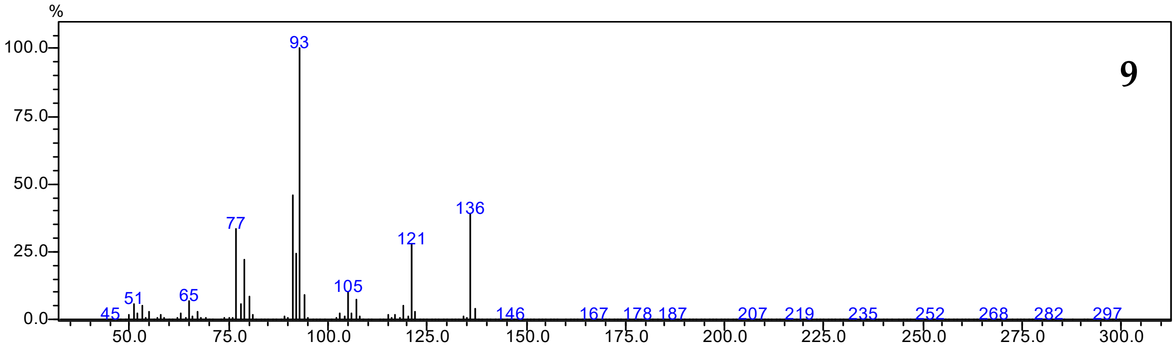
9
%
100.0
75.0
50.0
25.0
0.0
45 51 65 77 93 105 121 136 146 167 178 187 207 219 235 252 268 282 297
50.0 75.0 100.0 125.0 150.0 175.0 200.0 225.0 250.0 275.0 300.0

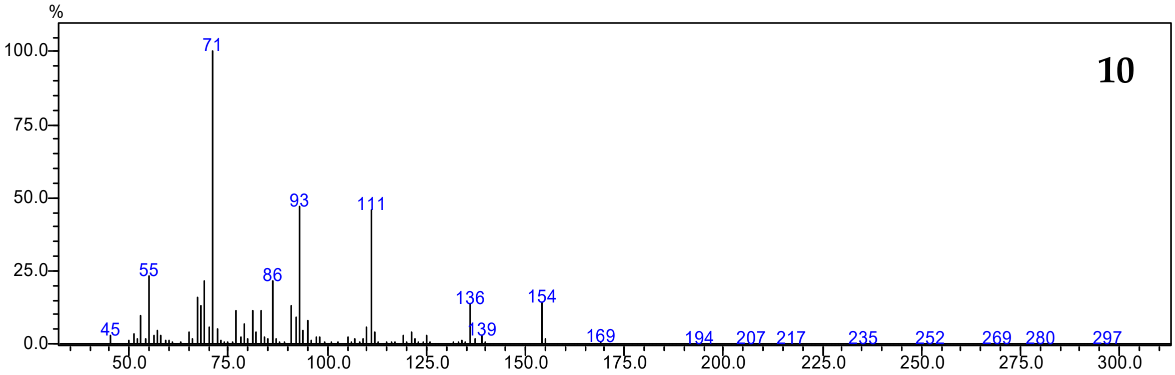
10
%
100.0
75.0
50.0
25.0
0.0
45 55 71 86 93 111 136 139 154 169 194 207 217 235 252 269 280 297
50.0 75.0 100.0 125.0 150.0 175.0 200.0 225.0 250.0 275.0 300.0

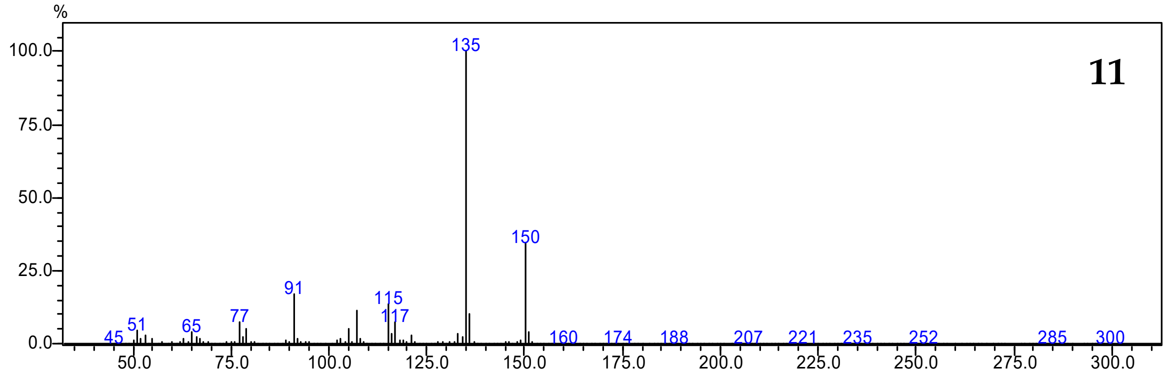

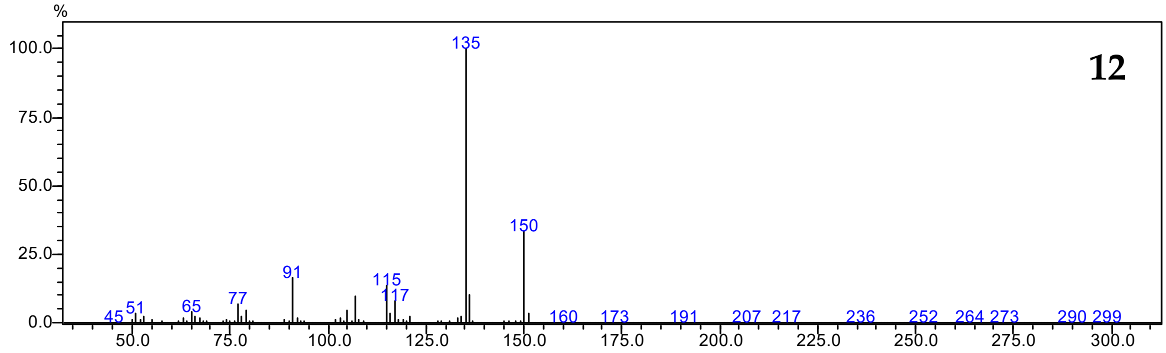

Figure S1. Copies of MS spectra of substances identified in PVEO using GC/MS. **(1)** α-Thujene, **(2)** α-Pinene, **(3)** Sabinene, **(4)** β-Pinene, **(5)** β-Myrcene, **(6)** (+)-4-Carene, **(7)** *m*-Cymene, **(8)** D-Limonene, **(9)** γ-Terpinene, **(10)** *p*-Menth-1-en-4-ol, **(11)** Thymol, **(12)** Carvacrol.

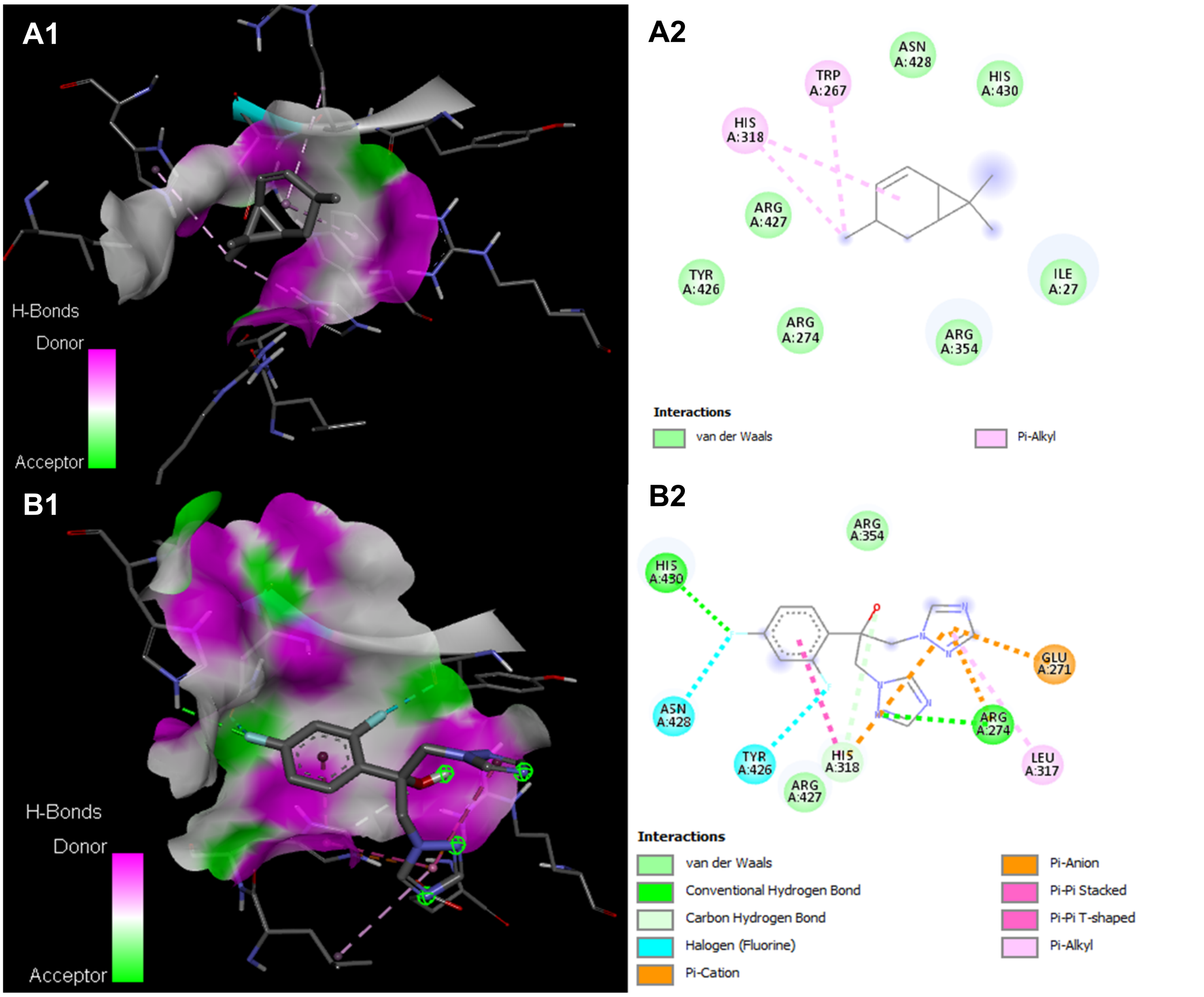

Figure S2. 2D, and 3D schemes of the interactions of 4-(+)-Carene (A1, and A2), the native ligand (Fluconazole) (B1, and B2), with the active site of Cytochrome P450 14 α-sterol Demethylase (PDB ID: 1EA1).

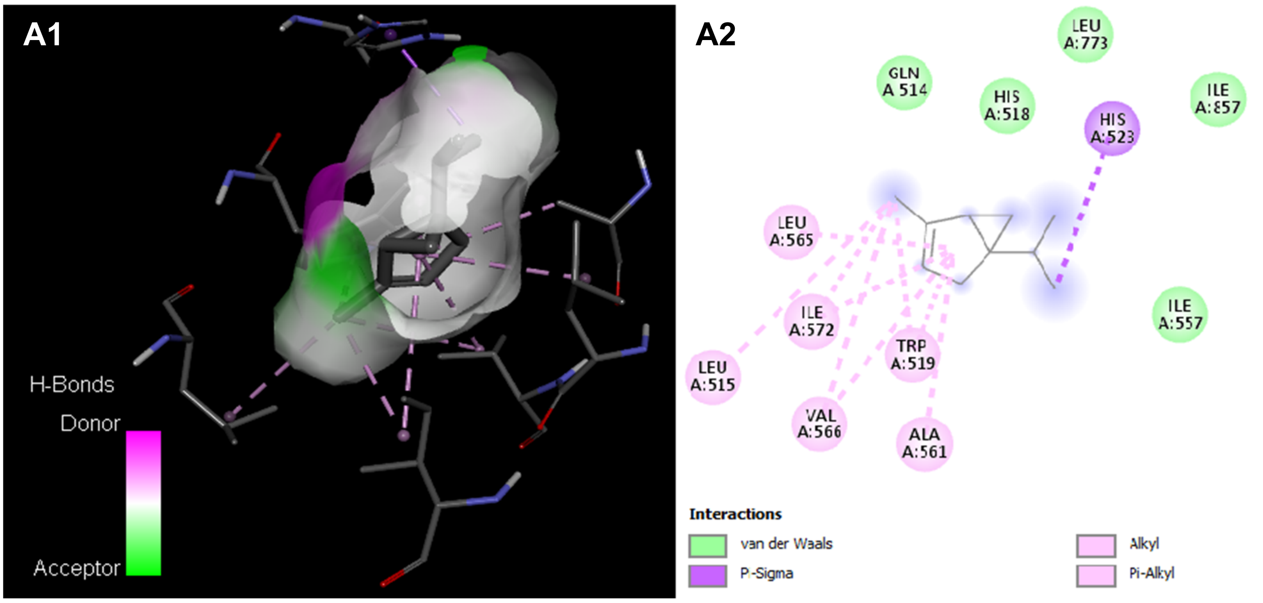

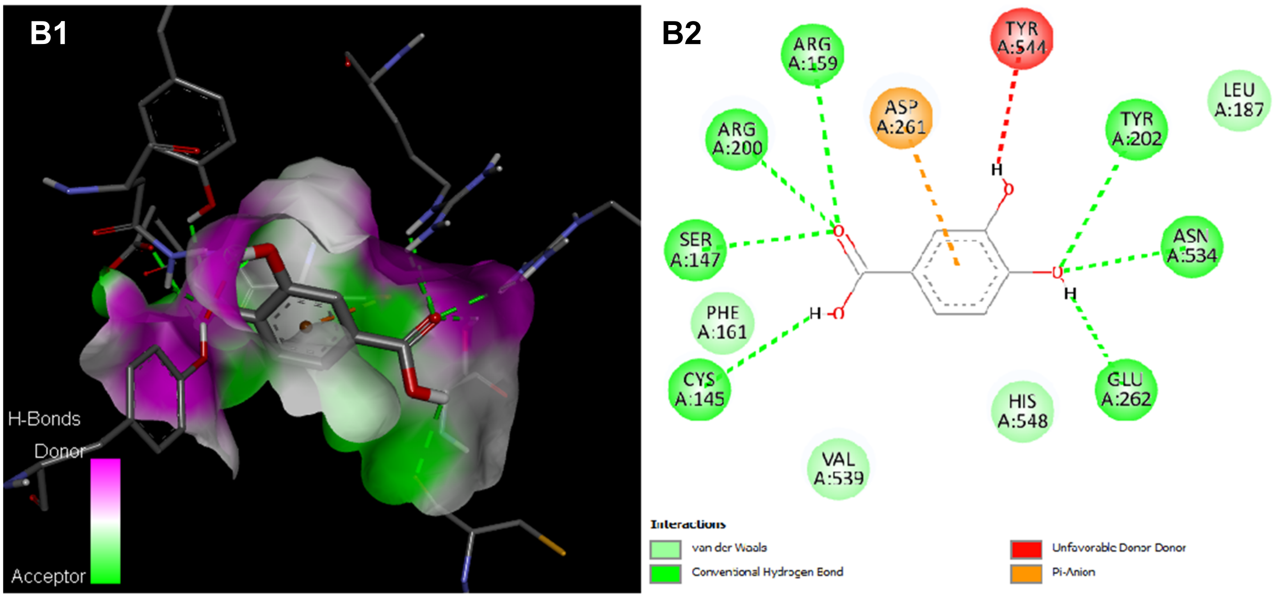

Figure S3. 2D, and 3D schemes of the interactions of α-Thujene (A1, and A2), the native ligand (Protocatechuic Acid) (B1, and B2), with the active site of lipoxygenase (PDB ID: 1N8Q)

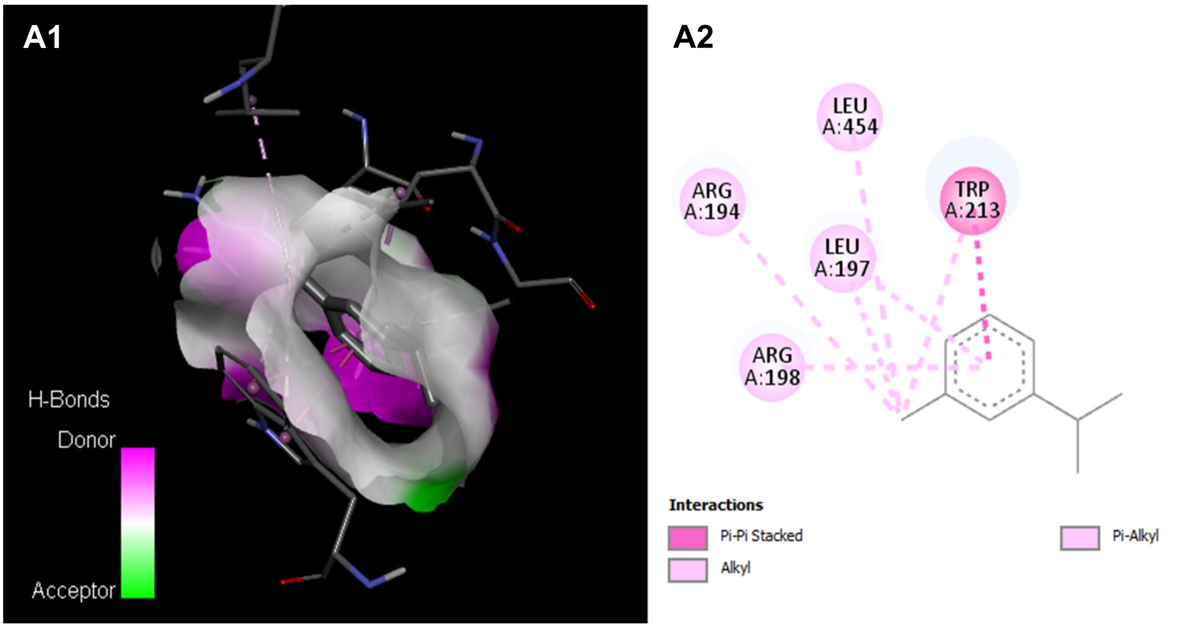

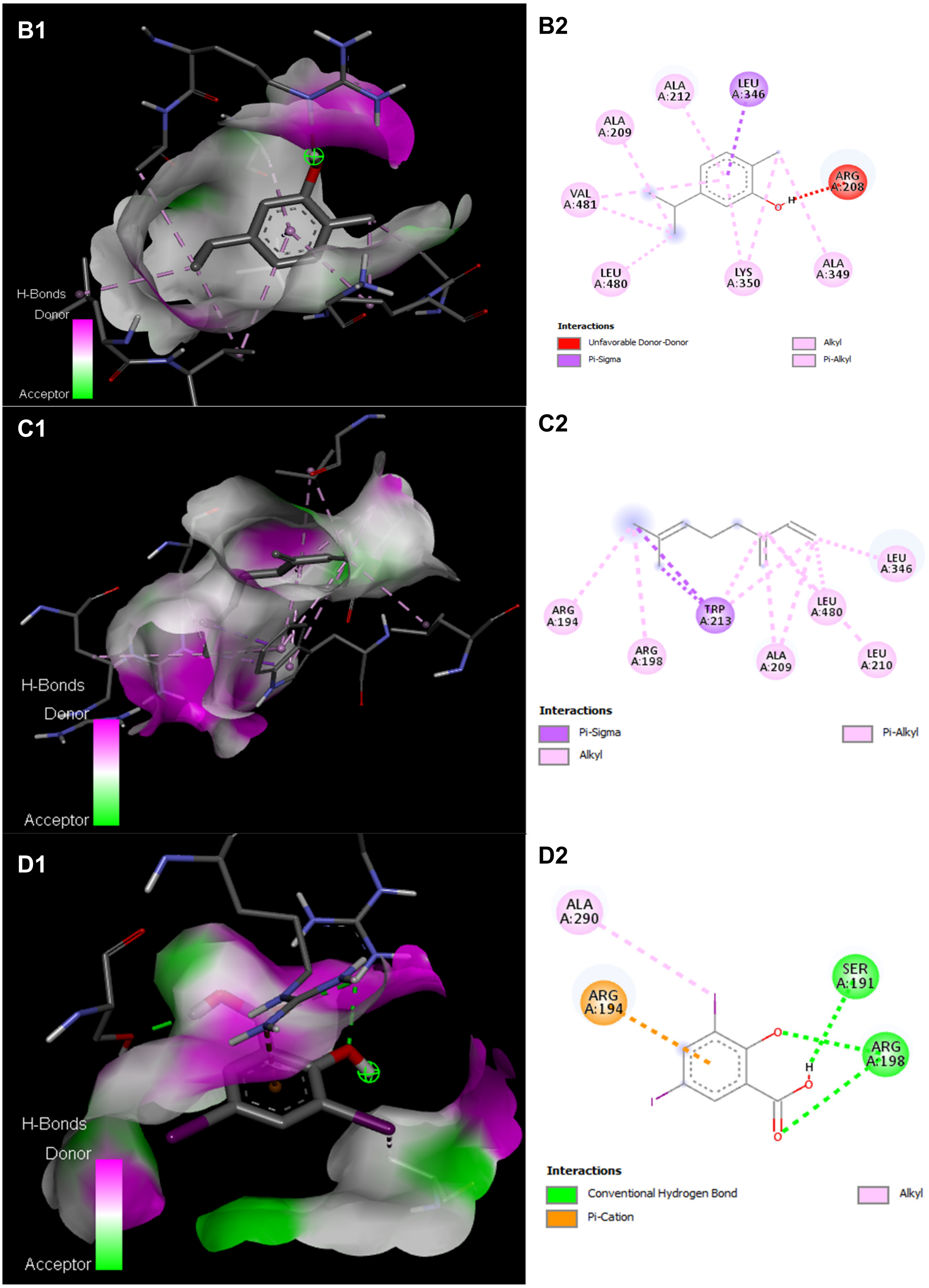

Figure S4. 2D, and 3D schemes of the interactions of *m*-Cymene (A1, and A2), Carvacrol (B1, and B2), β-Myrcene (C1, and C2), and the native ligand (3,5-Diiodosalicylic Acid) (D1, and D2), with the active site of bovine serum albumin (PDB ID: 4JK4)
